# Sen1p Contributes to Genomic Integrity by Regulating Expression of Ribonucleotide Reductase 1 (*RNR1*) in *Saccharomyces cerevisiae*

**DOI:** 10.1371/journal.pone.0064798

**Published:** 2013-05-31

**Authors:** Upendarrao Golla, Vikash Singh, Gajendra Kumar Azad, Prabhat Singh, Naveen Verma, Papita Mandal, Sakshi Chauhan, Raghuvir S. Tomar

**Affiliations:** Laboratory of Chromatin Biology, Department of Biological Sciences, Indian Institute of Science Education and Research, Bhopal, India; Florida State University, United States of America

## Abstract

Gene expression is a multi-step process which requires recruitment of several factors to promoters. One of the factors, Sen1p is an RNA/DNA helicase implicated in transcriptional termination and RNA processing in yeast. In the present study, we have identified a novel function of Sen1p that regulates the expression of ribonucleotide reductase *RNR1* gene, which is essential for maintaining genomic integrity. Cells with mutation in the helicase domain or lacking N-terminal domain of Sen1p displayed a drastic decrease in the basal level transcription of *RNR1* gene and showed enhanced sensitivity to various DNA damaging agents. Moreover, *SEN1* mutants [Sen1-1 (G1747D), Sen1-2 (Δ1-975)] exhibited defects in DNA damage checkpoint activation. Surprisingly, *CRT1* deletion in Sen1p mutants (Sen1-1, Sen1-2) was partly able to rescue the slow growth phenotype upon genotoxic stress. Altogether, our observations suggest that Sen1p is required for cell protection against DNA damage by regulating the expression of DNA repair gene *RNR1.* Thus, the misregulation of Sen1p regulated genes can cause genomic instability that may lead to neurological disorders and premature aging.

## Introduction

In eukaryotic organisms, genomic integrity is constantly challenged by various intrinsic and extrinsic genotoxic stresses, which is monitored by sophisticated cellular protein networks known as DNA damage response (DDR). Generally, eukaryotic cells respond to DNA damage by arresting the cell cycle and by inducing genes implicated in DNA damage repair [1]. In budding yeast, the Mec1/Rad53/Dun1 cell cycle checkpoint kinase pathway is required to slow down or arrest the progression in all phases of the cell cycle. At the same time, Mec1 and Rad53 induce the transcription of a number of DNA repair genes [2]. The *MEC1* and *RAD53* genes are evolutionarily conserved, which link DNA damage and checkpoint control to various human disorders and cancer [3]. Genes encoding ribonucleotide reductase (RNR) are the best-studied transcriptional targets of DNA replication and damage which are regulated by Mec1/Rad53 checkpoint kinase pathway [4], [5]. There are four RNR genes (*RNR1, RNR2, RNR3* and *RNR4*) known to encode DNA repair proteins which are highly inducible upon DNA damage.

The RNR complex is comprised of two major subunits (Rnr1, Rnr3) and two minor subunits (Rnr2, Rnr4) [6], [7]. The RNR complex catalyzes the rate-limiting step in the production of deoxyribonucleoside triphosphates (dNTPs) from ribonucleotides which are in turn precursors for DNA synthesis [8]–[10]. Among the four RNR genes, *RNR2*, *RNR3*, and *RNR4* are repressed by Crt1 (Constitutive RNR transcription 1) [2]. The fourth RNR gene *RNR1* is not controlled by Crt1, however it is also DNA damage-inducible [11]. In absence of DNA damage, the sequence-specific DNA binding protein Crt1 binds to upstream repression sequences (URS) of the *RNR3* and represses transcription after recruiting the Ssn6-Tup1 co-repressor complex to the promoter [2]. Transcriptional activation of RNRs is regulated by Mec1/Rad53 checkpoint-dependent phosphorylation and the accompanying release of Crt1 from the promoter [2]. In addition to transcriptional regulation, the activity of RNR complex is inhibited at the post-translational level by Sml1 (suppressor of mec1 lethality) which binds to Rnr1p [12]. RNR activity is high during S-phase and increases after DNA damage to elevate dNTPs level [13], [14]. Similarly, RNR activity is increased after DNA damage by Dun1-dependent phosphorylation of the Rnr1 inhibitor, Sml1p [15].

In addition to regulation of RNR by transcription and protein kinase cascades, TOR (target of rapamycin) plays a central role in DNA damage response. TOR is a member of the phosphatidylinositol 3-kinase-related kinase family which regulates cellular responses to a wide-range of environmental stresses, including nutrient starvation, growth factor deprivation, and hypoxia. Also, TOR signalling is required for replication fork progression and to maintain elevated levels of Rnr1 and Rnr3 induced by Rad53 checkpoint activation [16]. Expression of RNRs is highly dependent on the recruitment of Polymerase-II, TBP, TBP associated factors (TAFs), chromatin remodelling and modifying complexes on their promoters [17], [18]. Recently, it has been shown that the telomere binding protein ‘Repressor activator protein 1 (Rap1)’ is required for the activation of RNR genes [19].

There are several classes of DNA damage repair factors such as helicases, transcription factors and chromatin modifiers. In *Saccharomyces cerevisiae*, *SEN1* gene codes for a 252 kDa protein (Sen1p) that localizes to nucleus and is essential for growth. According to sequence similarity and functional characterization of the *S. pombe* ortholog (sen1), *S. cerevisiae* Sen1p is believed to be an ATP-dependent RNA/DNA helicase. However, the helicase activity has not been shown directly [20]. Sen1p is originally known for transcriptional termination, processing of small nuclear RNAs and defence against oxidative DNA-damage [21]–[23]. Sen1p is present in a multi-protein interactome and is subjected to post-translational modifications like phosphorylation and acetylation which may be essential for the regulation of transcription, RNA processing and genomic integrity [24]–[27]. Sen1p functions through specific interactions with the largest subunit of RNA polymerase II (Rpb1p) and Rnt1p which is a key component of the RNA-processing machinery [26]. Mutation in *S. cerevisiae SEN1* gene causes accumulation of tRNA, ribosomal RNA precursors and some small nuclear RNAs [21]. It is evident that mutations in human Senataxin SETX (human ortholog of yeast Sen1)), ortholog of yeast Sen1p causes neurological disorders such as Ataxia-Occulomotor Apraxia (AOA) and Juvenile Amyotrophic Lateral Sclerosis (ALS) [28]–[30]. Global analysis of protein complexes in yeast revealed that Sen1p may exist in complex with a wide-variety of proteins like histones [31], Rnr1 [31] and Dun1 [32]. However, experimental evidences for these interactions are lacking. Synthetic growth defect and two-hybrid analysis revealed the interaction of Sen1p with Srs2 which is a DNA helicase involved in check-point activation and DNA damage response [27], [33]. Many of the mutations in human SETX have been localized at the N-terminal region of the protein which is essential for protein-protein interactions. Upon reconstruction of some of the human *SETX* mutations in yeast *SEN1*, it has been shown to affect specific protein-protein interactions such as the ability of Sen1p to bind to the C-terminal domain of Rpb1p [26]. Due to the above rationale; we decided to investigate the role of Sen1p in gene expression by taking DNA repair genes as a model.

In the present study, we have identified a novel functional role of Sen1p in the regulation of *RNR1* gene. We observed a decrease in basal level expression of *RNR1* in cells lacking the N-terminal region (Δ1-975) or carrying a mutation in the helicase domain (G1747D) of Sen1p. We were partly able to rescue slow growth phenotype of the Sen1p mutant by deleting *CRT1*. In addition, mutations in Sen1p exhibited defects in Rad53 phosphorylation, which suggests the probable role of Sen1p in the activation of checkpoint kinase pathway upon genotoxic stress. Taken together, our studies indicate that Sen1p has a novel role in maintaining genomic integrity by regulating the expression of *RNR1* and that mutations in Sen1p can lead to the misregulation of DNA repair genes which is the fundamental basis of several diseases including neurological disorders.

## Results

### Sen1p Localizes to the Nucleus in both Free and Chromatin Bound Forms

**Figure 1:**
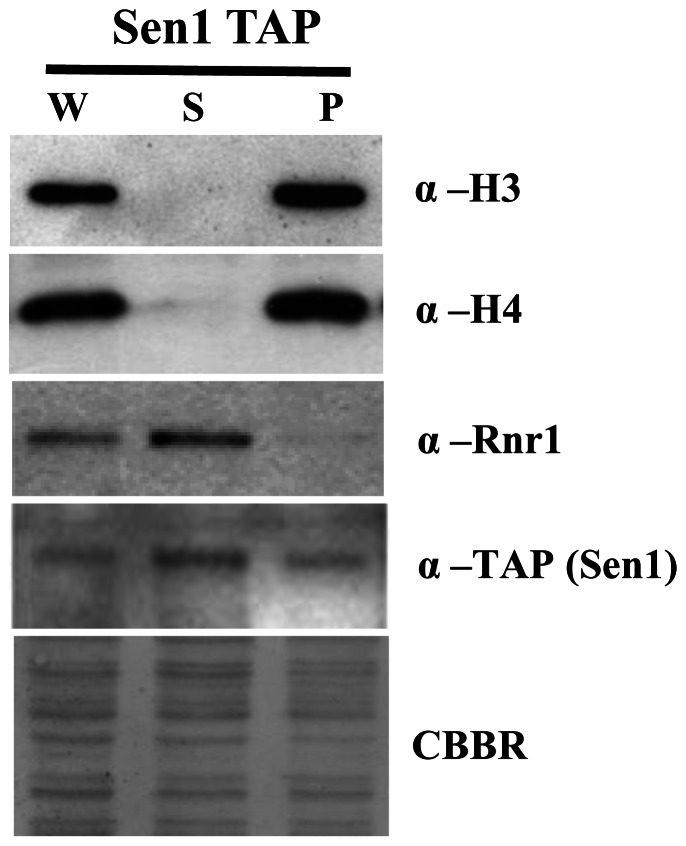
Sen1p is localized to nucleus, exists in both chromatin bound and unbound form. **B)** Chromatin association assay; WT (Sen1-TAP) strain was grown in regular YPD and whole cell extract (W), supernatant chromatin unbound (S) and pellet (P) chromatin enriched fractions were analyzed by western blotting. Antibodies used are shown on the right side of the figure. CBBR stands for Coomassie Brilliant Blue R.

Several studies in yeast and human cells revealed that protein complexes redistribute in response to genotoxic stress [34]–[37]. Sen1p of *S. cerevisiae* is a known component of the NRD complex (Sen1, Nab3 and Nrd1) implicated in the transcription termination of non-polyadenylated as well as some polyadenylated RNA polymerase II transcripts [38], [39]. We performed a chromatin association assay for analyzing the possibility of Sen1p associating with chromatin. The purity of chromatin enriched pellet (P) and soluble (S) fraction was examined by western blot analysis using antibodies against chromatin bound proteins (Histone H3 & H4) and chromatin non-interacting proteins (Ribonucleotide reductase, Rnr1). Interestingly, our chromatin association assay results revealed that Sen1p exists in both chromatin bound and free forms (Fig. 1).

### N-terminal Truncation (Δ1-975) of Sen1 Leads to Slow Growth Phenotype

**Figure 2:**
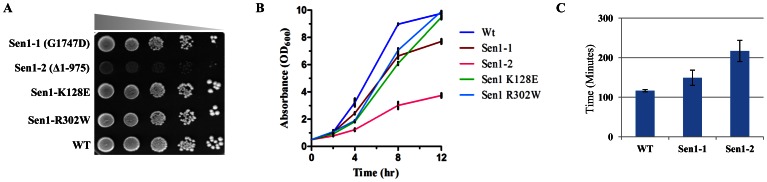
N-terminal truncation (Δ1-975) of Sen1 leads to slow growth phenotype. A**)** Growth Assay**;** WT (Sen1TAP) and the different mutants of Sen1 [Sen1-1(G1747D), Sen1-2(Δ1-975), Sen1(K128E), Sen1(R302W)] were grown up to log-phase. 3 µl of each undiluted and 10-fold serially diluted cultures were spotted on control YPDA plate. B**)** Growth of Sen1p strains was recorded in liquid YPD media and analyzed in comparison to wild-type cells. The average cell density (OD_600_) of two independent isolates of Sen1p strains with error bars was plotted against different time points (0, 2, 4, 8 and 12 hr). C**)** The doubling time for different Sen1p strains was calculated three times as described in materials and methods, represented in minutes.

Interaction of Sen1 with several protein factors suggests that Sen1 is involved in the regulation of multiple nuclear processes and is essential for growth. The N-terminal truncation of (Δ1-975) Sen1 resulted in delayed growth phenotypes in plating assay (Fig. 2A), which were readily apparent in liquid culture by growth curve analysis (Fig. 2B). The loss of the N-terminal domain of Sen1 resulted in an increase in the doubling time (Fig. 2C), suggesting probable delay in cell cycle progression and other cellular pathways.

### Sen1p is Required for Maintenance of Basal *RNR1* Levels

**Figure 3:**
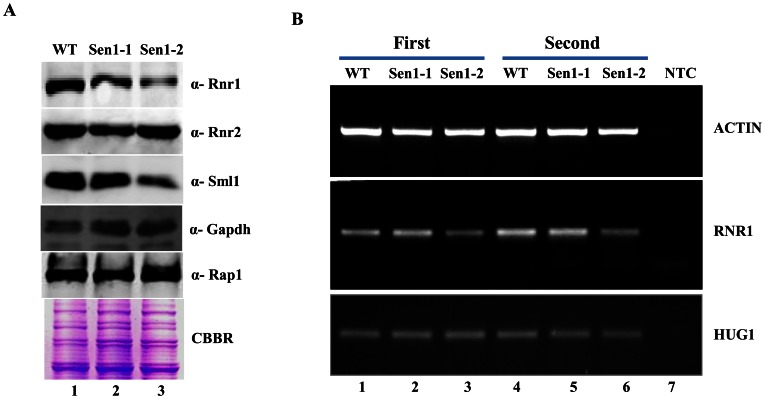
Sen1 is required to maintain basal levels of *RNR1* through transcription. **A)** Untreated WT, Sen1-1 and Sen1-2 cells were grown in liquid YPD to mid-log and used for making whole cell protein extracts by TCA precipitation. Samples were analyzed by western blotting with Rnr1, Rnr2, Sml1, Rap1 and Gapdh antibodies. CBBR stands for Coomassie Brilliant Blue R.**B)** Semi-quantitative analysis of *RNR1, HUG1* genes; logarithmically grown Sen1 strains (WT, sen1-1, Sen1-2) in YPD were left untreated for 3 hr. Total RNA was extracted and reverse transcribed to cDNA. Semi-quantitative PCR analysis was performed to assess the levels of *RNR1, HUG1* and *ACT1* transcripts. The expected sizes of the PCR product for *RNR1, HUG1* and *ACT1* are 219, 190, and 520 bp respectively. Two repeats of RT-PCR amplifications are shown here.

Functional Sen1p is required for the maintenance of genomic integrity. We suspected that the slow growing phenotypes exhibited by Sen1-2(Δ1-975) might be due to genomic instability. To uncover the molecular rationale behind the delayed growth phenotypes of sen1 mutant, we analyzed the expression profiles of *RNRs*. Surprisingly, our results showed a reduction in both, protein as well as mRNA basal levels of *RNR1* (Fig 3A and 3B). Rnr1 is an essential larger subunit of the RNR complex, which plays a major role in the maintenance of cellular basal dNTP levels. So, the defect in the basal *RNR1* levels may account for the resultant phenotypes.

### Sen1p Mutants are Hypersensitive to TOR Inhibitor

**Figure 4:**
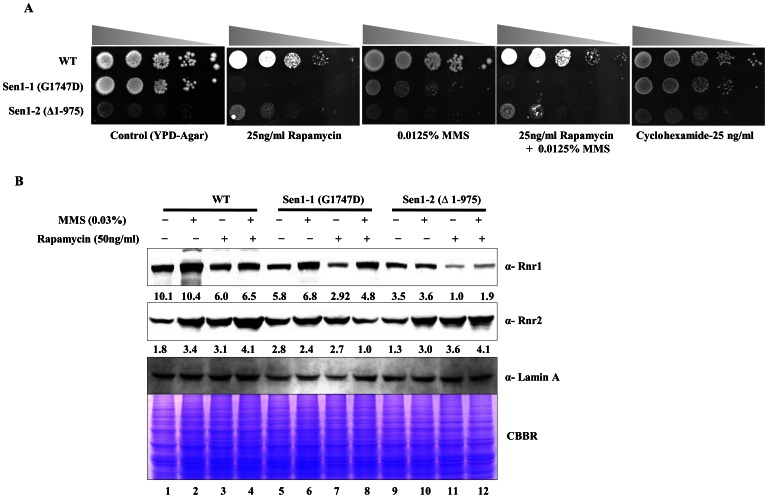
Sen1p mutants are sensitive to Rapamycin. **A)** Growth assay; Sen1 wild-type, Sen1-1 and Sen1-2 strains were grown at 30°C to log-phase. 3 µl of each undiluted and 10-fold serially diluted cultures were spotted on control YPDA or YPDA plates containing Rapamycin (25 ng/ml), MMS (0.125%), Cyclohexamide (25 ng/ml) and combination of both Rapamycin (25 ng/ml) & MMS (0.125%). Photographs were taken after 72 hr of incubation at 30°C. **B)** Wild-type (Sen1-TAP), Sen1-1, Sen1-2 strains were grown to log-phase in liquid YPD. The culture of cells was divided equally and then left untreated or treated with either of Rapamycin (50 ng/ml), MMS (0.03%) or both Rapamycin (50 ng/ml) & MMS (0.03%) for 3 hr. Rnr1 and Rnr2 protein levels in wild type and sen1 mutants were analyzed by western blot using Rnr1, 2 specific antibodies (Lamin A antibody was used as loading control). Quantification of intensity of each band were performed by using Image J software, relative value of band intensity for Rnr1 and Rnr2 western signal are mentioned below each lane after normalising with Lamin A intensity. CBBR stands for Coomassie Brilliant Blue R.

The TOR pathway regulates cellular responses to a wide-variety of stresses, including nutrient starvation and hypoxia. Recent studies revealed the functional role of TOR signalling for cell survival in response to genotoxic stress [16]. We thus tested if TOR signalling inhibition by rapamycin (RAP) could affect the Rnr1 levels in Sen1 mutants. We performed sensitivity assays using Sen1 mutants on solid YPDA plates containing RAP, MMS and both. Interestingly, we observed that a mutation in the helicase domain (Sen1-1) leads to hypersensitivity towards RAP (Fig 4A). To ensure that this observation was not simply an artefact of translation inhibition by RAP, we performed a spot assay in the presence of Cyclohexamide (CHX), a global protein translation inhibitor. The Sen1p mutants didn’t exhibit sensitivity to CHX, which confirmed our hypothesis that sensitivity of Sen1 mutants to TOR inhibitor was due to the RAP-induced additional decrease in Rnr1 level. To further substantiate our observation, we analyzed the Rnr1 and Rnr2 protein levels in Sen1 mutants (Sen1-1 and Sen1-2) upon treatment with RAP, MMS and both. Rapamycin alone was able to reduce Rnr1 level in WT, Sen1-1 and Sen1-2 (Fig 4B). These results suggest that the slow growing phenotype observed in Sen1-1 and Sen1-2 is due to a decrease in basal level expression of Rnr1 and this defect can be further enhanced by inhibiting TOR signalling.

### Sen1-1 and Sen1-2 Exhibited Partial Resistance to Genotoxic Stress upon CRT1 Deletion

**Figure 5:**
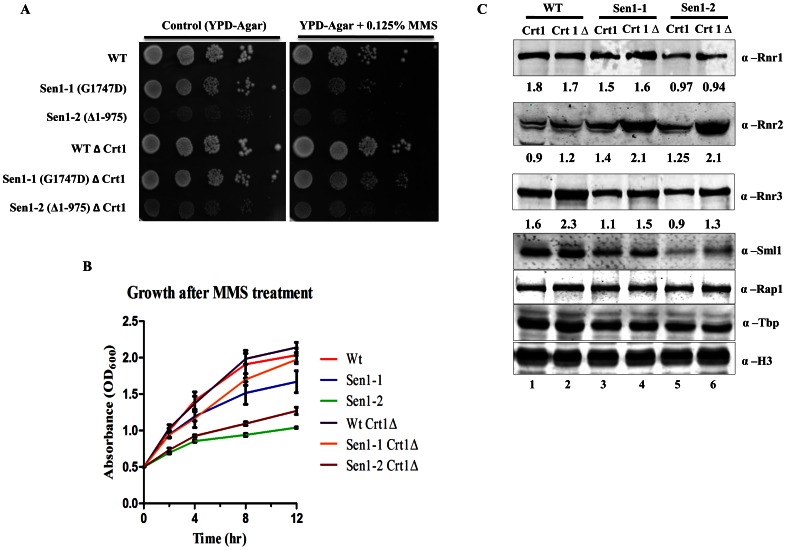
Sen1p mutants upon *CRT1* deletion develop resistance to DNA damage. **A)** Growth assay; Growth rate of Sen1 strains with or without Crt1was analyzed by spot test on solid YPDA medium. Sen1-TAP (wt), Sen1-1 and Sen1-2 were grown at 30°C to log-phase. 3 µl of each undiluted and 10-fold serially diluted cultures were spotted on control YPD or YPD containing 0.125% MMS. Photographs were taken after 48 hr of incubation at 30°C. **B)** Growth of WT (Sen1-TAP), Sen1-1 and Sen1-2 strains with and without CRT1 was recorded in the presence MMS (0.03%). The average cell density (OD_600_) of two independent isolates with error bars was plotted against different time points (0, 2, 4, 8 and 12 hr). **C)** Sen1-TAP (WT), Sen1-1, Sen1-2 with and without *CRT1* deletion strains were grown in liquid YPD medium. Whole cell extracts were made by TCA precipitation and extracts were analyzed by western blotting using antibodies as shown. Experiment is performed in triplicate and one of the representative figure is shown here. Quantification of intensity of each band were performed by using Image J software, relative value of band intensity for Rnr1, Rnr2 and Rnr3 western signal are mentioned below each lane. Band intensity is normalised with respect to Rap1 western signal.

Defect in basal level of Rnr1 expression in Sen1-1 and Sen1-2 mutants led us to propose that the slow growth phenotype might be due to a decrease in dNTP levels. Synthesis of dNTPs is catalyzed by the RNR complex [40], [41]. In *S. cerevisiae*, the *CRT1* gene encodes a DNA-binding protein Crt1 which binds to the upstream repression sequences (URS) of *RNR 2* & *3*. Upon DNA damage, Crt1 is phosphorylated by the Mec1p-Rad53p-Dun1p kinase pathway which leads to the release of Crt1 [2]. As there was a defect in transcription of basal *RNR1* levels in Sen1-1 and sen1-2 mutants, we used *crt1Δ* cells to recover slow growing phenotypes upon genotoxic stress. The Sen1p mutants showed partial resistance to MMS after Crt1 deletion (Fig. 5A). Results from the growth assay in the presence of MMS on solid media (Fig. 5A) are also apparent in liquid media (Fig. 5B), and these results account for the constitutive induction (derepression) of *RNR2* & *RNR3* (Fig. 5C) after Crt1 deletion. These results suggest that an increased expression of Rnr2 & Rnr3 (Fig. 5C) upon Crt1 deletion compensates for *RNR1* expression, hence exhibiting resistance against genotoxic agents.

### Mutations in Sen1p Lead to Sensitivity Towards DNA Damaging Agents

**Figure 6:**
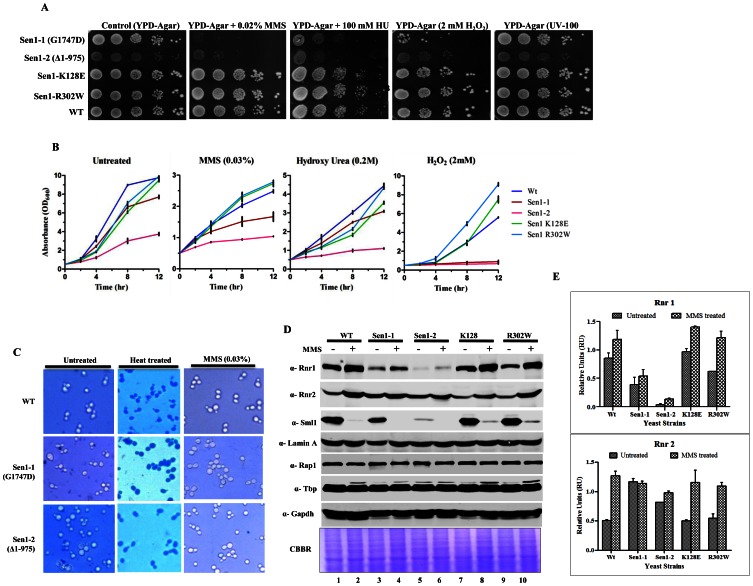
Mutations in Sen1p cause sensitivity to DNA damaging agents. **A)** Growth Assay**;** Sen1-TAP (Wild-type) and the different mutants of Sen1 [(Sen1(G1747D), Sen1-2(Δ1-975), Sen1(K128E), Sen1(R302W)] were grown up to log-phase. 3 µl of each undiluted and 10-fold serially diluted cultures were spotted on control YPDA or YPDA plates containing 0.02% MMS, 100 mM HU, 2 mM H_2_O_2_ and one set exposed to UV of 100 J/m^2^. All plates were incubated at 30°C for 3 days and photographed. **B)** Growth of Sen1 strains was recorded in the presence of different agents like MMS (0.03%), Hydroxy Urea (0.2 M), H_2_O_2_ (2 mM) and analyzed in comparison to untreated (control) cells. The average cell density (OD_600_) of two independent isolates of Sen1 strains with error bars was plotted against different time points (0, 2, 4, 8 and 12 hr). **C)** Viability Assay; Sen1-TAP (Wt), Sen1-1 and sen1-2 strains were cultured up to mid log phase and treated with 0.03% MMS for 12 hr and cells were stained with 0.3% methylene blue for checking viability. Untreated and heat killed cells were taken as negative and positive controls respectively; viability was observed under light microscope (40X) and photographed. **D)** MMS (0.03%) treated and untreated Sen1-TAP (WT), Sen1-1, Sen1-2, Sen1(K128E) and Sen1(R302W) cells were used for making whole cell protein extracts by TCA precipitation, samples were analyzed by western blotting. Rnr1, Rnr2, Rnr3 antibodies were used to examine the expression of ribonucleotide reductase proteins and Sml1. **E)** Quantification of Rnr proteins (Rnr1, 2 & 3) level in Sen1 strains from Fig.4A before and after 3 h of MMS (0.03%) treatment. ‘Lamin A’ was used as the internal control (refer materials and methods). Error bars represent standard error of the mean (SEM).

Mutations in Sen1p affect the basal level of Rnr1 and genomic integrity. This motivated us to unveil the probable role of Sen1p in DNA damage response. To investigate the role of Sen1p in DNA damage response, we examined the effect of different DNA damaging agents (MMS, HU, H_2_O_2_ and UV) on Sen1p mutants. Sen1p mutant alleles Sen1-1 and Sen1-2 displayed enhanced sensitivity to DNA damaging agents in both solid media and liquid culture (Fig. 6A and 6B). Furthermore, individual mutations in Sen1p confer differential sensitivity towards different DNA damaging agents. These results suggest an apparent role of Sen1p in DNA damage response through distinct mechanisms, either through modulation of DNA replication or the endogenous nucleotide pool (Fig. 6A). Certainly, cells with point mutations in the N-terminal domain of Sen1 (K128E, R302W) are not sensitive to DNA damaging agents in comparison to Sen1-1 & Sen1-2 (Fig. 6A). These findings indicate that Sen1p dependent cellular functions and its interactome are essential for survival during DNA damage. Subsequently we performed a cell viability assay by staining cells with a vital dye, methylene blue, to assess whether the sensitivity of Sen1p mutant strains to MMS was due to cell-cycle arrest or cell death. We used untreated cells as negative control (no staining) and heat treated cells as positive control (dark blue stained) (Fig. 6C). Interestingly, MMS sensitive Sen1p mutants were not stained by the vital dye. This observation suggests that the sensitivity of Sen1p mutants to MMS is probably due to cell-cycle arrest or defects in progression but not cell mortality.

### Sen1-1 and Sen1-2 Mutants Induce Rnr1 and Rnr2 upon Genotoxic Stress

Since Sen1-1 and Sen1-2 displayed higher sensitivity to DNA damaging agents, we analyzed the expression of DNA damage inducible factors. Expression of ribonucleotide reductases (RNRs) is highly inducible upon DNA damage [8], [9], [42]. The expression of Rnr proteins (Rnr1 and Rnr2) was examined in Sen1p strains by western blot analysis with or without MMS treatment (Fig. 6D). Expression levels of Rnr proteins were quantified using ImageJ software as described in materials and methods (Fig. 6D). The above results showed conventional induction of Rnr1, 2 proteins after MMS treatment in Sen1p mutants. But the level of Rnr1 induction in Sen1-2 was proportional to the reduced basal levels, which further suggested the necessity of Sen1p for the maintenance of cellular basal level of Rnr1. In response to DNA damage, Sml1p, an inhibitor of RNR complex, is phosphorylated by Dun1p and subsequently degraded to increase the enzymatic activity of the RNR complex [43], [44]. Our western blot analysis revealed that there was significant degradation of Sml1p upon MMS treatment in all Sen1 mutants, although the basal level of sml1p was significantly less in untreated Sen1-1 and Sen1-2 in comparison to wild type cells.

We reasoned that the decrease in the basal levels of Rnr1 in Sen1-1 and Sen1-2 strains (Fig. 6D- lanes 3, 5) was most probably due to degradation shown in earlier studies [45], [46]. To investigate if either vacuole or proteasome-dependent pathways promoted the degradation of Rnr1 & 2 protein levels after MMS treatment, we analyzed MMS-induced Rnr protein levels in the presence of a vacuolar inhibitor (PMSF) or a proteasome inhibitor (MG132). Pre-incubation of cells with PMSF or MG132 for 60 minutes followed by MMS treatment did not alter the levels of observed Rnr1 proteins (figure S1). This result led us to propose that the failure to observe induction of Rnr proteins in Sen1-1 and Sen1-2 after MMS treatment was regulated independent of protein degradation pathways. Altogether, these results led us to propose that the sensitivity of Sen1-1and Sen1-2 to MMS may account for the reduced Rnr1 basal levels.

### Contribution of Sen1p to Checkpoint Activation in MMS Treated Cells

**Figure 7:**
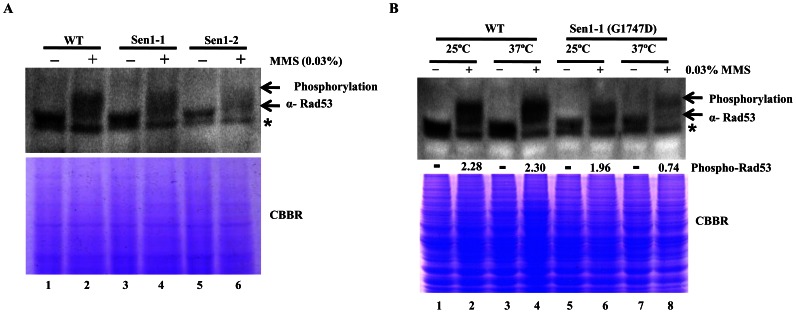
Mutations in Sen1p affects Rad53 phosphorylation states upon genotoxic stress. **A)** Phosphorylation of Rad53 was analyzed in Sen1 strains upon genotoxic stress with MMS. Sen1-TAP (WT), Sen1-1 and Sen1-2 were grown to log-phase and then left either untreated or treated with MMS (0.03%) for 3 hr at 25°C. Untreated and MMS treated cells were used for making whole cell extract by TCA method and samples were analyzed by western blotting using Rad53 antibody. **B)** Phosphorylation of Rad53 was analyzed in Wild-type (Sen1-TAP), temperature sensitive mutant Sen1-1 at 25°C and 37°C. Whole cell extracts were prepared from WT, Sen1-1 cells grown to log-phase at 25°C and 37°C by TCA precipitation. Samples were analyzed by western blotting with Rad53 antibody. CBBR stands for Coomassie Brilliant Blue R.

In *Saccharomyces cerevisiae*, the DNA damage response is activated by the Mec1-Rad53-Dun1 kinase pathway to intercede cell-cycle dependent checkpoint activation, to activate DNA repair and to promote the regulation of RNR activity. The MMS sensitive phenotypes of Sen1-1 and Sen1-2 mutants led us to check for phosphorylation of Rad53, which is a vital transducer kinase in the activation of DNA damage checkpoint. A hallmark of checkpoint activation is a shift in Rad53 towards hyperphosphorylated state, differentially depending on the type of genotoxic stress [47]. We examined whether mutations in Sen1p affect Rad53 activation in comparison to wild type cells after MMS treatment. We detected obvious checkpoint alteration in Sen1 mutants (Sen1-1 & Sen1-2) by analyzing Rad53 phosphorylation (Fig. 7A). Upon MMS treatment the Rad53 mobility change corresponds to the shift from intermediate to full activation in WT cells (Figure 7A – lane 2). We found considerable defect in Rad53 phosphorylation upon MMS treatment in Sen1-1 and Sen1-2 in comparison to WT cells (Fig 7A lanes 2, 4, 6). The earlier genetic studies on Sen1p carrying a temperature-sensitive mutation (Sen1-1) indicate that loss of Sen1p function results in various pleiotropic defects in transcript processing, termination, RNA processing and genomic stability [27], [38], [48], [49]. So, we were motivated to examine checkpoint activation by Rad53 in Sen1-1 cells under both permissive (25°C) and non-permissive (37°C) temperatures after MMS treatment (Fig. 7B). Interestingly, Sen1-1 mutant showed a modest disappearance of the Rad53 intermediate state at non-permissive temperature when compared to permissive temperature (Fig. 7B – lanes 6, 8) after MMS treatment. But, WT cells didn’t exhibit any significant change in Rad53 phosphorylation at both permissive and non-permissive temperatures after MMS treatment. More specifically, we envisaged that the delayed activation of Rad53 may correlate with the sensitivity of Sen1-1 and Sen1-2 to MMS treatment. Taken together, the above results suggested the probable contribution of Sen1p in DNA damage checkpoint activation and consequently repair.

## Discussion

The *Saccharomyces cerevisiae SEN1* gene codes for Sen1p, a nuclear-localized ATP-dependent DNA/RNA superfamily I helicase. *SEN1* is an ortholog of human SETX (senataxin), which has been implicated in neurological disorders like AOA and ALS [29], [30]. Mutations in *SEN1* resulted in pleiotropic phenotypes defective in transcriptional termination, RNA processing and DNA repair, and have deleterious effects on genomic integrity. Moreover, Sen1p is embedded in a complex of protein-protein interactions and displays genetic interaction with DNA damage repair genes [21], [26], [27]. However, the functional importance of these *SEN1* interactions in the regulation of gene expression and DNA damage response remains unexplored. In this study, our focus on functional characterization of Sen1p demonstrated a novel role for the normal expression of RNR1 gene and for checkpoint activation during DNA damage response. In the absence of DNA damage, the mutation in Sen1 leads to a significant decrease in *RNR1* expression. *RNR* inhibitor Sml1, the levels of which too are controlled by the Mec1-Rad53-Dun1 pathway, is down-regulated in sen1 mutants. Our subsequent studies on Sen1p mutants showed an enhanced sensitivity to DNA damaging agents thereby pointing out its role in DNA-damage response.

Sen1p is 2231 amino acid residues long and is essential for growth. Although, the first 975 N-terminal amino acids of Sen1p are dispensable for growth, they are shown to interact with Rbp1, Rnt1p, Rad2p and SmD3p [26]. Earlier studies showed that the loss of Sen1 function resulted in various pleiotropic defects in transcript processing and termination [22], [50]. In our study, the Sen1-2 mutant displayed delayed-growth phenotype showing a noteworthy increase in doubling time, indicating that this domain performs various cellular functions required for normal growth (Fig. 2D–E). Our results are consistent with earlier observations which demonstrated that Sen1p might play role in the regulation of cell-cycle upon phosphorylation by Cdk1 kinase [24] and acetylation by NatB (N-acetyltransferase) [25]. Also, Sen1p has been shown to interact with another NatB substrate, Glc7p [51] which is an important regulator of energy metabolism, cell wall integrity, G2/M cell cycle progression and morphogenesis [52]–[54].

Recently it has been reported that a mutation affecting the function of Sen1 helicase led to significant alterations in polymerase II distribution over non-coding and protein-coding genes, suggesting that Sen1p has an important role in the regulation of gene expression [55]. Earlier studies have shown that mutations in *SEN1* lead to genome instability linked to the presence of RNA/DNA hybrids [56]; hence we proposed that the delayed growth phenotype of the Sen1-2 mutant might be due to genomic instability. To validate our proposal, we have checked the level of Rnr proteins (Rnr1, Rnr2) which are known to be induced during genomic instability. To our surprise, Sen1-1 and Sen1-2 strains had shown a decrease in basal levels of Rnr1 and Sml1p (an inhibitor of Rnr1) (Fig. 3A). In addition, we detected decreased mRNA levels of *RNR1* in Sen1 mutants (Fig. 3B). Rnr1 is an essential member of the RNR complex and it is required for catalyzing dNTP synthesis. Under normal growth conditions, Sml1 remains associated with Rnr1 to inhibit the formation of the active RNR complex which is necessary for synthesis of dNTPs [57]. Over-expression of RNR1 leads to the up-regulation of Sml1p [58]. Interestingly, we observed a reduction in Sml1p levels in Sen1 mutants which further supported the reduced levels of Rnr1. The decreased Rnr1 levels provide an explanation for the slow growth phenotype displayed by Sen1 N-terminal truncation (Sen1-2). All four *S. cerevisiae* RNR genes are activated by DNA damage and replication blocks. The pathway involved in the activation of *RNR2, RNR3* and *RNR4* is well understood and requires the Mec1-Rad53-Dun1 kinase cascade, which targets Crt1p, a transcriptional inhibitor of DNA-damage-inducible genes. Earlier, it has been shown that *RNR1* expression does not depend on Crt1p [11]. Thus, the downstream Dun1-Crt1 part of the Mec1-Rad53-Dun1-Crt1 pathway is not involved in the regulation of *RNR1*.

Under normal condition, *CRT1* deletion causes constitutive expression (derepression) of *RNR2, RNR3* and *RNR4* genes [59]. Temperature-sensitive alleles of *RNR1* can be rescued by over expression of *RNR3* at non-permissive temperature [60]. As Sen1-1 and Sen1-2 cells showed reduced Rnr1 levels, we next asked whether over-expression of Rnr3 can rescue the delayed growth phenotype of Sen1-1 and Sen1-2 mutants upon genotoxic stress. Rnr3 is known for increasing the efficiency of RNR complex. Surprisingly, we were able to partly rescue the slow growth phenotype of Sen1 mutants upon genotoxic stress by MMS after *CRT1* deletion (Fig. 5A–B). The sensitivity of Sen1 mutants to rapamycin further supported our hypothesis that Sen1p regulates the expression of *RNR1* (Fig. 4). Rapamycin is known to regulate RNR levels through the inhibition of TOR pathway. TOR is a highly conserved serine/threonine kinase that functions as a central regulator of eukaryotic cell responses to environmental stresses, such as amino acid starvation, hypoxia, and growth factor deprivation [61], [62]. Additionally, our findings revealed the role of Sen1p in nutritional stress, which is mimicked by RAP treatment. The drastic reduction in Rnr1 level (Fig. 4B, lanes 5 and 7) in the presence of rapamycin may explain the hypersensitivity of Sen1-1 on plate assays (Fig. 4A). The Sen1-2 mutant was found to be less sensitive for rapamycin compared to Sen1-1 mutant (Fig. 4A), while we observed a similar pattern of reduction in rnr1 level upon rapamycin treatment in both mutants (Fig 4B, compare lanes 5 and 7, lanes 9 and 11). In continuation to earlier studies showing proteomic and genetic interactions of SEN1 with DNA damage repair factors, we investigated its role in DNA damage response. Interestingly, Sen1-1 and Sen1-2 exhibited sensitivity to various DNA damaging agents like MMS, HU and H_2_O_2_ (Fig. 6A), but they were able to induce Rnr1 even though basal levels of Rnr1 was significantly less compared to wild type cells upon MMS treatment (Fig. 6D). These observations further supported that Sen1 is required for maintaining the basal level expression of *RNR1*.

Rad53 plays a central role in DNA damage response by regulating the induction of RNR genes by downstream signalling of the kinase cascade upon genotoxic stress. Loss of Rad53 leads to multiple defects, including inability to recover from replication blocks, excess histone accumulation, X-ray sensitivity and impaired checkpoint activation resulting in slow growth and chromosome loss [63]–[65]. Rad53 is phosphorylated at more than 20 serine or threonine residues, some of which are phosphorylated differentially depending on the type of genotoxic stress [47], [66]. Some site-specific modifications, such as T-loop phosphorylation, are necessary for Rad53 activation in all situations. We observed obvious checkpoint alterations in Sen1 mutants compared to wild type cells, corresponding to the failure in shift of phosphorylated Rad53 from intermediate to full activation (Fig. 7B). In support of the above observation, our analysis of checkpoint activation in Sen1-1 at non-permissive temperature showed a dramatic decrease in Rad53 phosphorylation (Fig. 7B). Since Rad53 lies upstream of RNR1, we hypothesise that Sen1p acts on Rad53 to affect downstream events such as RNR1 activation. Earlier, it has been shown that RNR1 expression does not depend on Crt1 [4], [67]. Thus, the downstream Dun1-Crt1 part of the Mec1-Rad53-Dun1-Crt1 pathway, which is known to control the DNA-damage-inducible genes in yeast, is not involved in the regulation of Rnr1. Alternatively, it is also possible that Sen1p is acts independently on Rad53 leading to a defect in Rnr1 levels. Cumulatively, the findings suggest the probable contribution of Sen1p to checkpoint activation in DNA damage response.

Recently it has been shown that human Senataxin (SETX) co-localizes with 53BP1, a key DNA damage response protein, and also with other factors involved in DNA repair [68]. There are many examples which demonstrate the link between defects in DNA repair proteins and neurodegenerative syndromes [69]. In addition, mutations in DNA damage signalling kinase ATM have been linked to Ataxia-telangiectasia, whereas Ataxia-telangiectasia-like disorder (ATLD) and Nijmegen breakage syndrome (NBS) are caused by mutations in two components of the MRN complex, MRE11 and NBS1 respectively. Nonetheless, those aforementioned examples reveal the importance of known components of DNA damage response in double strand break (DSB) repair. Our findings in yeast further demonstrate that Sen1p plays an important role in maintaining genomic integrity by regulating *RNR1* gene and proper DNA damage checkpoint activation. Although we have provided strong genetic and biochemical evidences to support that Sen1p is involved in the transcriptional regulation of RNR1 gene, its role in the recruitment of transcription factors and chromatin remodelling machinery remains to be studied. Basic knowledge about the function of SEN1 through studies in yeast would presage future efforts to understand the mechanism of specific gene regulation defects associated with *SEN1* mutations.

## Materials and Methods

### Strains, Chemicals, Growth Media and Growth Conditions

The *Saccharomyces cerevisiae* used in this study are listed in Table S1. *CRT1* deleted yeast strains were constructed by PCR-mediated gene disruption [70], [71] method using primers listed in Table S2. Gene replacement was confirmed by the absence and presence of *CRT1* and *RNR1* coding regions respectively by PCR amplification using genomic DNA from Δcrt1 cells as template. Media components, chemicals and all other molecular biology grade reagents used in this study were purchased from Sigma–Aldrich, Merck, Himedia, GE Healthcare, Invitrogen, New England Biolabs, Thermo Scientific, and R&D systems. Unless stated otherwise, yeast cells were grown at 30°C in YPD (yeast extract 1%, peptone 2%, and dextrose 2%) liquid media. For solid YPDA media, 2% Bacto-agar was used in addition to YPD [72].

### Growth Assay

To investigate the effect of various DNA damaging agents on the growth of Sen1 yeast strains, spot assay was carried out using serial dilutions of mid-log phase cultures. Three micro-litres of each undiluted and 10-fold serially diluted cultures were spotted onto solid YPDA plates without and with geotaxis agents like MMS (0.02%) or HU (100 mM). To assess the effect of hydrogen peroxide (H_2_O_2_) on growth, early-log phase cells were subjected to 2 mM H_2_O_2_ for 3 hr. Each of undiluted and 10-fold serially diluted untreated or H_2_O_2_ stressed cultures were spotted onto YPDA medium. One set of YPDA plates spotted with untreated cells was immediately exposed to Ultraviolet (UV) radiation of 100 J/m^2^ using Ultraviolet Cross linker (CL-1000) chamber. For TOR inhibition study, serially diluted log-phase cultures were spotted on to YPDA plates supplemented with Rapamycin (25 ng/ml) or with Cyclohexamide (25 ng/ml). All the plates were incubated at 30°C and growth was recorded at time intervals of 24, 48 and 72 hr by scanning plates using HP scanner.

### Growth Curves, Microscopy and Viability Assay

Yeast strains were grown in regular YPD liquid medium as above to an absorbance (OD_600_) of 0.5. Cultures were either left untreated (control) or were treated with MMS (0.03%), HU (0.2 M), and H_2_O_2_ (2 mM). The absorbance (OD_600_) of untreated and treated cultures was measured at 2, 4, 8 and 12 hr time intervals subsequent to the treatments. Growth curves were plotted with average cell density (absorbance-OD_600_) of two independent repeats against different time points. Also the doubling time of WT and different mutants were calculated using the formula:

**Doubling time** = t/g [Where, t = the time cultured; g = [log10 (N_t_/N_0_)]/0.3; N_0_ = Number of cells or OD_600_ at start, N_t_ = Number of cells or OD_600_ at the end].

Small aliquots of yeast cultures after 12 hr treatment were placed onto slides and were visualized under a LEICA DM500 microscope (installed with the LAS EZ-V1.7.0 software) to record the morphology of yeast cells. Cells were analyzed for viability after 12 hr of MMS (0.03%) treatment by staining with 3.7% buffered methylene blue [73]. Dark blue stained cells were metabolically inactive or nonviable. Heat treated cells for 10 min at 70°C were used as a positive control. As expected, 100% of heat killed cells stained dark blue.

### Preparation of Protein Extracts, Electrophoresis and Immunoblotting Analysis

Yeast mid-log phase cultures were treated with 0.03% MMS for 3 hr. After 3 hr, cells were harvested and frozen. Whole cell protein extracts were obtained from the frozen yeast cell pellets with 20% trichloro acetic acid (TCA) precipitation following a standard protocol [74], [75]. The protein extracts were separated by SDS-polyacrylamide gel electrophoresis. Protein gels were stained with Coomassie Brilliant Blue R-250 (CBBR) followed by destaining with methanol & acetic acid and photographed. For immunoblotting analysis, extracts were transferred onto PVDF membranes using a Bio-Rad mini wet transfer apparatus (Bio-Rad, USA) and were then blocked with Odyssey blocking buffer (LI-COR® Biosciences) for 45 min followed by sequential incubations with primary and secondary antibodies for 1 hr. IRDye® 800CW anti-Rabbit IgG or anti-Mouse IgG were used as secondary antibody. Blots were scanned by using Odyssey Infrared imager (LI-COR® Biosciences). Following primary antibodies were used: General H3 (Sigma, H0164), H3K4me1 (Abcam, 8895), H3K4me2 (Abcam, 32356), H3K27me2 (Abcam, 24684), H3K36me3 (Sigma, SAB4800028), H3K9Ac (Abcam, 69830), H3K18ac (Cell signaling, 9675), H3K23ac (Abcam, 61234), H3K27ac (Abcam, 45173), H3K9acS10ph (Sigma, H9161), H4K8ac (Abcam, 15823), H4K16ac (Abcam, 61240), Rnr1 (Agrisera, ASO9 576), Rnr2 (Agrisera, ASO9 575), Rnr3 (Agrisera, ASO9 574), Sml1 (Agrisera, AS10 847) GAPDH (Abcam, 37168), Lamin A (Abcam, 26300), Rad53 (Santa Cruz Biotechnology Inc., SC-6749), TAP (Thermo Scientific, CAB1001), RNA Pol II (Abcam, ab817). Polyclonal antibodies against recombinant TBP and RAP1 were generated by immunization of rabbits. Fuji gel-dock system (LAS-4000 mini) was also used for detection some of the western signals by chemiluminescence. Expression of Rnr proteins (Rnr1, Rnr2 & Rnr3) in Sen1 strains with and without MMS (0.03%) treatment was measured by quantifying the protein levels using ImageJ software (http://rsbweb.nih.gov/ij). Expressions were quantified from three independent repeats, represented as relative units (RU, levels of Rnr protein divided by the levels of Lamin A in corresponding sample).

### Chromatin Association Assay

Chromatin association assay was performed as described previously with certain modifications [76]. Briefly, yeast cells from 50 ml mid-log phase culture were washed with pre-spheroplasting buffer (100 mM Tris-HCl pH 8.8, 10 mM DTT). Spheroplasts were prepared by incubating in Spheroplasting buffer (50 mM Tris-HCl pH 7.5, 0.6 M Sorbitol, 10 mM DTT) containing 250 U lyticase at 37°C for 15 min. Spheroplasts were washed once with ice-chilled wash buffer (100 mM KCl, 50 mM HEPES-KOH pH 7.5, 2.5 mM MgCl_2_, and 0.4 M Sorbitol). Following extraction in buffer (EB; 100 mM KCl, 50 mM HEPES-KOH pH 7.5, 2.5 mM MgCl_2_, 1 mM DTT, 1 mM PMSF and protease inhibitor cocktail), spheroplasts were lysed by adding Triton X-100 to 1% and incubating on ice for 5 min with gentle mixing. 25% of suspension was kept as whole cell extract (WCE) and rest was underlayered with 50% volume of 30% sucrose (volume refers to the volume of spheroplast suspension in EB), and centrifuged at 12,000 rpm for 10 min at 4°C. The supernatant (S) was collected as chromatin unbound fraction and the pellet (chromatin bound fraction) was washed with 25% volume of EB containing 0.25% Triton X-100 (EBX), and centrifuged at 10,000 rpm for 5 min at 4°C. Equal volume of 2X SDS loading dye was added to each of supernatant (S) and pellet (P) fractions. Samples were boiled for 5 min and analyzed by western blotting with indicated antibodies.

### Transcriptional Analysis by RT-PCR

The yeast cells were grown up to mid-log phase at 30°C. The cells were harvested by centrifugation and total RNA was extracted by heat/freeze phenol method and treated with DNase I as described earlier with slight modification [77]. 2 µg of total RNA was reverse transcribed to synthesize cDNA using High Capacity RNA-to-cDNA™ Kit (Life Technologies Corporation, California) according to the manufacturer instructions. The cDNA was diluted 10 times and then used for PCR reaction using primers of *RNR1*, *HUG1* and *ACT1*. The oligonucleotides used in this study were listed in Table S2.

### Protein Degradation Analysis

Rnr proteins degradation was measured in the presence of either the proteasome inhibitor MG132 (Sigma) or the vacuolar inhibitor PMSF (Sigma). WT, Sen1-1 and Sen1-2 cultures were grown at 30°C to 5×10^6^ cells/mL in YPD media, were split and pre-incubated with either 100 µM MG132 or 1 mM PMSF and vehicle [dimethyl sulfoxide (DMSO)] for 90 min prior to treatment with MMS (0.0125%) as described earlier [45]. The cells were harvested after 60 min of treatment with MMS. Whole cell protein extracts were obtained as described above using 20% TCA and subjected to western blot analysis.

## Supporting Information

Figure S1 **Defect in Rnr1 levels in Sen1p mutant is independent of protein degradation.** Wild-type (WT), sen1-1, sen1-2 strains were grown to log-phase and culture of cells was divided equally and pre-incubated with either 1 mM PMSF or 100 mM MG132 for 90 minutes, and then treated with MMS (0.0125%) for 60 minutes. Rnr1 and Rnr2 protein levels in wild type and sen1 mutants were analyzed by western blot using Rnr1, 2 and 3 specific antibodies, Lamin A antibody used as loading control. The Rnr protein levels were quantified and represented as Relative Units (RU) as described in materials and methods.
(TIF)

Table S1 **List of yeast strains used in this study.**
(DOCX)

Table S2 **List of oligonucleotide primers used in this study.**
(DOCX)
