# Supplementary figures and images for: Sen1p Contributes to Genomic Integrity by Regulating Expression of Ribonucleotide Reductase 1 (RNR1) in Saccharomyces cerevisiae
Source: PLoS One. 2013 May 31;8(5):e64798. doi: 10.1371/journal.pone.0064798 (PMC3669351; doi:10.1371/journal.pone.0064798)

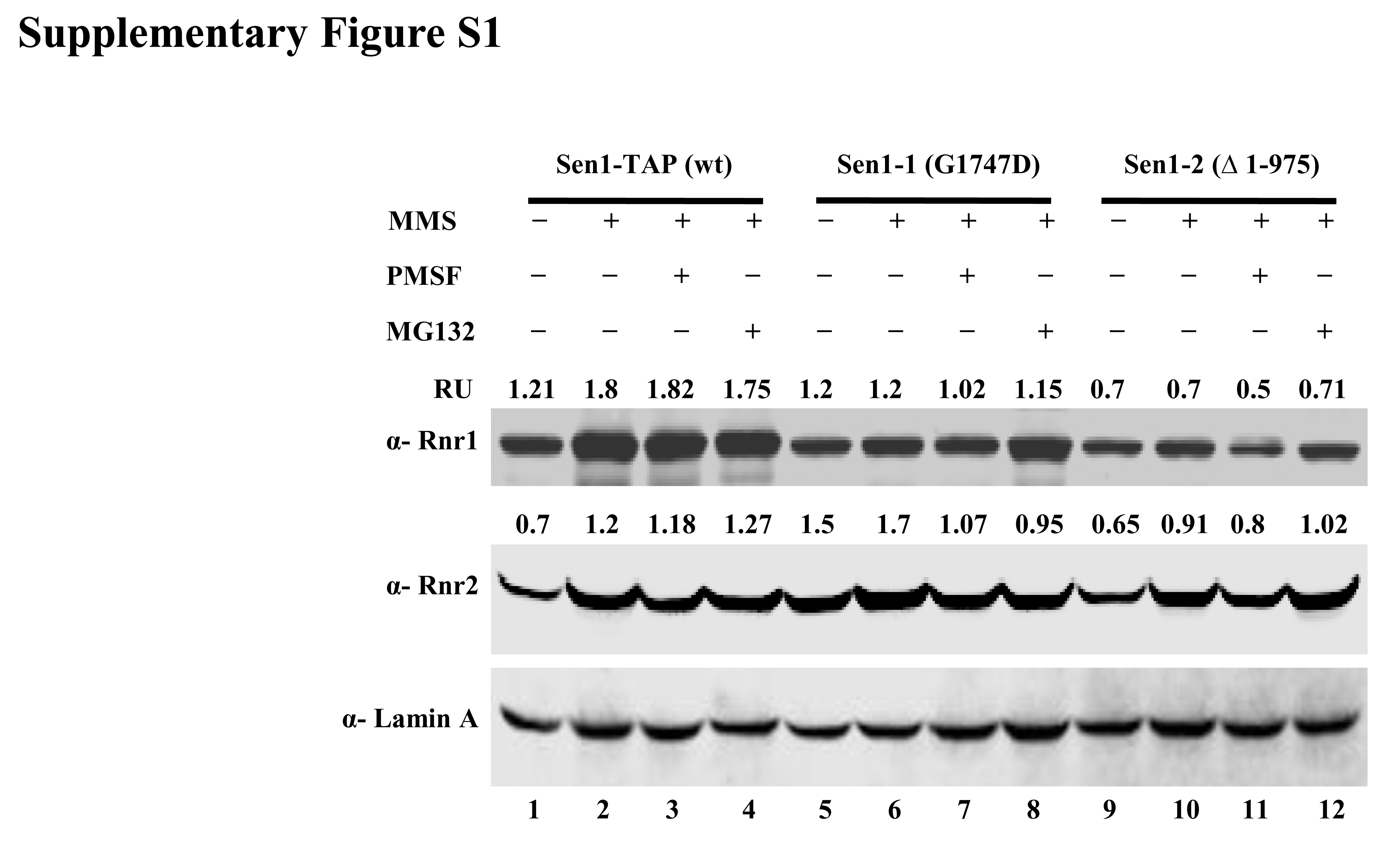

Supplement: Figure S1 — Defect in Rnr1 levels in Sen1p mutant is independent of protein degradation. Wild-type (WT), sen1-1, sen1-2 strains were grown to log-phase and culture of cells was divided equally and pre-incubated with either 1 mM PMSF or 100 mM MG132 for 90 minutes, and then treated with MMS (0.0125%) for 60 minutes. Rnr1 and Rnr2 protein levels in wild type and sen1 mutants were analyzed by western blot using Rnr1, 2 and 3 specific antibodies, Lamin A antibody used as loading control. The Rnr protein levels were quantified and represented as Relative Units (RU) as described in materials and methods. (TIF) [file pone.0064798.s001.tif]
