# Supplementary material for: Sen1p Contributes to Genomic Integrity by Regulating Expression of Ribonucleotide Reductase 1 (RNR1) in Saccharomyces cerevisiae
Source: PLoS One. 2013 May 31;8(5):e64798. doi: 10.1371/journal.pone.0064798 (PMC3669351; doi:10.1371/journal.pone.0064798)
Supplement: Table S1 — List of yeast strains used in this study. (DOCX) [file pone.0064798.s002.docx]

Supplementary Table 1. List of Strain used in this study.

| S.No. | Strain | Genotype |
| --- | --- | --- |
| 1 | WT | MAT**a** leu2Δ0 ura3Δ0 his3Δ1 met15Δ0 SEN1-TAP |
| 2 | Sen1-1 (G1747D) | Derived from FWY1(MATa ura3-52 leu2-3, _112 pep4-3 trp1 sen1-1  (Ursic et al. 2004) |
| 3 | Sen1-2 (Δ1-975) | Derived from DDY86 (MATa ade2-101 his3-200 lys2-801 trp1-D1 ura3-52 leu2-D1T sen1-2) (DeMarini et al. 1992) |
| 4 | Sen1-K128E | Derived from JFY41 (MATa leu2D ura3D his3D1 trp1D sen1-K128E)  (Finkel et al. 2010) |
| 5 | Sen1-R302W | Derived from DUY1513 (MATa leu2D ura3D his3D1 met15D sen1-R302W)  (Finkel et al. 2010) |
| 6 | YRS009 | WT, Crt1 Δ :: KanMX4 **(this study)** |
| 7 | YRS010 | Sen1-1 (G1747D), Crt1 Δ :: KanMX4 **(this study)** |
| 8 | YRS011 | Sen1-2 (Δ1-975), Crt1 Δ :: KanMX4 **(this study)** |
