# Supplementary material for: Sen1p Contributes to Genomic Integrity by Regulating Expression of Ribonucleotide Reductase 1 (RNR1) in Saccharomyces cerevisiae
Source: PLoS One. 2013 May 31;8(5):e64798. doi: 10.1371/journal.pone.0064798 (PMC3669351; doi:10.1371/journal.pone.0064798)
Supplement: Table S2 — List of oligonucleotide primers used in this study. (DOCX) [file pone.0064798.s003.docx]

**List of oligonucleotides used in this study.**

| **S.No** | **Oligonucleotide primers** | **Sequence(5'–3')** |
| --- | --- | --- |
| 1 | CRT1 (DP*) F | TGGCGATTTGGGAAAAAGTTGAAAAAAAAAATAGCAGTAACGGATCCCCGGGTTAATTAA |
| 2 | CRT1 (DP*) R | ATTCTTTTTTAAATATCCCCATATACTAATGATAGAACTTGAATTCGAGCTCGTTTAAAC |
| 3 | CRT1F | CCTCTGTGTCTATCGAATGCA |
| 4 | CRT1R | AGGCCATTGACGAATTTCATG |
| 5 | RNR1F | ATTTCGTGCCCGCAGC |
| 6 | RNR1R | TTCCTCATCATCAACGATGGG |
| 7 | HUG1F | CCTTAACCCAAAGCAATTCTTCC |
| 8 | HUG1R | TTAGTTGGAAGTATTCTTACCAATGTC |
| 9 | ACT1F | CACCCTGTTC TTTTGACTGAAGC |
| 10 | ACT1R | TACCGGCAGATTCCAAACCC |

***Deletion Primer (DP)**
